# Isolation and characterization of an anti-proliferative polysaccharide from the North American fungus *Echinodontium tinctorium*

**DOI:** 10.1038/s41598-022-21697-0

**Published:** 2022-10-14

**Authors:** Mehreen Zeb, Wai Ming Li, Christian Heiss, Ian Black, Linda E. Tackaberry, Hugues B. Massicotte, Keith N. Egger, Kerry Reimer, Parastoo Azadi, Chow H. Lee

**Affiliations:** 1 Department of Chemistry and Biochemistry, Faculty of Science and Engineering, University of Northern British Columbia, Prince George, BC V2N 4Z9 Canada; 2 Department of Ecosystem Science and Management, Faculty of Environment, University of Northern British Columbia, Prince George, BC V2N 4Z9 Canada; 3 Complex Carbohydrate Research Center, University of Georgia, Athens, GA 30602 USA

**Keywords:** Cancer, Plant sciences

## Abstract

A novel polysaccharide EtGIPL1a was purified from fruiting bodies of *Echinodontium tinctorium*, a fungus unique to western North America. EtGIPL1a has an estimated weight average molecular weight of 275 kDa and is composed of glucose (54.3%), galactose (19.6%), mannose (11.1%), fucose (10.3%), glucuronic acid (4%), and rhamnose (0.6%). It has multiple glycosidic linkages, with 3-Glc*p* (28.9%), 6-Glc*p* (18.3%), 3,6-Glc*p* (13%), 4-Glc*p*A (9.2%), 6-Gal*p* (3.9%), 2,6-Gal*p* (2.6%), 3-Fuc*p* (2.5%), 6-Man*p* (2.4%) being the most prominent, and unsubstituted glucose (15.3%), mannose (1.3%) and fucose (0.9%) as major terminal sugars. EtGIPL1a has a backbone containing mostly 3-substituted *β*-glucopyranose with 4-substituted glucopyranosyluronic acid. EtGIPL1a showed anti-proliferative activity against multiple cancer cell lines, with IC_50_ ranging from 50.6 to 1446 nM. Flow cytometry analyses confirmed that apoptosis induction is one mechanism for its anti-proliferative activity. EtGIPL1a should be further investigated for its potential anti-cancer activity in animal models, and for its possible utility in differentiation cancer therapy.

## Introduction

*Echinodontium tinctorium* (Ellis & Everh.) Ellis & Everh., is a hydnaceous perennial mushroom species belonging to the Echinodontiaceae family and Russulales order. It is unique to western North America and is widely distributed in the British Columbia (Canada) interior and coastal temperate rainforests. Nine species were historically recognized in the genus *Echinodontium* but some of these have subsequently been reassigned to other genera, or require re-collection and verification. Therefore, the genus is currently comprised of *E. tinctorium*, *E. tsugicola*, *E. ryvardenii* and the rare *E. ballouii*^1^.

*Echinodontium tinctorium* grows as a wood decay parasitic fungus on mature hemlock (*Tsuga*) and true fir (*Abies*) tree species, and produces a characteristic hoof-shaped, red-pigmented toothed conk^2^. It has a distinct color and was historically used by Indigenous peoples to create red paint pigments^3^. Previous studies on *E. tinctorium* have mainly focused on its taxonomy, phylogeny and symbiotic relationships^1^. Until recently, it had never been explored for potential medicinal properties and bioactive compounds. A polysaccharide with anti-inflammatory activity in vitro and in mice, termed AIPetinc, was isolated from the alkali extract of *E. tinctorium*^4^. In addition, an immuno-stimulatory polysaccharide containing glucuronic acid, called EtISPFa, was isolated from the water extract of *E. tinctorium*^5^.

Small molecules have also been isolated from *E. tinctorium* and related species. A lanostane derivative, echinodol, and a fluorone pigment, echinotinctone, were isolated from *E. tinctorium*^3,6^. Other small molecules isolated from species related to *E. tinctorium* include: echinolactone A and echinolactone B from *E. japonicum*^7^, now called *Echinodontiellum japonicum*^1^, and tsugicolines A–E^8^, echinodone, deacetyl-echinotinctone, 3-epiechinodol, and deacety-l-3-epiechinodol^9^, all from *E. tsugicola*. As yet, these compounds have not been investigated for potential medicinal properties.

In this study, we hypothesized that *E. tinctorium* has anti-proliferative activity against cancer cells. *E. tinctorium* was sequentially extracted using 80% ethanol followed by 50% methanol. The methanol extract was phase separated and assessed for anti-proliferative activity using the cytotoxic MTT assay. We show, for the first time, that the methanol extract of *E. tinctorium* has anti-proliferative activity against cancer cells. The subsequent objective of this study was to purify and characterize the anti-proliferative compound from the methanol extract of *E. tinctorium*; this was determined to be a polysaccharide. We subjected the extract to multiple chromatographic steps (Sephadex LH-20, DEAE-Sephadex, Sephacryl S-500 HR, and HPLC BioSEC-3 chromatography) for purification. Structural analyses were performed. These included gas chromatography–mass spectrometry (GC–MS) to determine the monosaccharide content and glycosidic linkages, Fourier transform infrared (FTIR) spectroscopy to identify functional groups and, lastly, NMR analyses to determine the constitution and monosaccharide sequence of the polysaccharide. In summary, our results reveal a novel anti-proliferative polysaccharide from *E. tinctorium* and suggest that this mushroom species be considered medicinal.

## Results

### Isolation of EtGIPL1a from *Echinodontium tinctorium*

Powdered *E. tinctorium* was sequentially extracted using the following solvents: 80% ethanol, 50% methanol, water and 5% sodium hydroxide. Earlier work indicated that only the 50% methanol extracts exhibited anti-proliferative activity against HeLa human cervical cancer cells^10^. Here, we aimed to purify, identify and characterize the anti-proliferative compound(s) from *E. tinctorium*. Supplementary Fig. S1a online summarizes the scheme used to obtain the methanolic extract 1B, which was then subjected to chloroform extraction. The aqueous phase L1 obtained, as well as the crude methanol extract 1B, were lyophilized and assessed for anti-proliferative activity. As shown in Supplementary Fig. S1b online, both 1B and L1 displayed dose-dependent anti-proliferative effect on HeLa cells.

**Figure 1:**
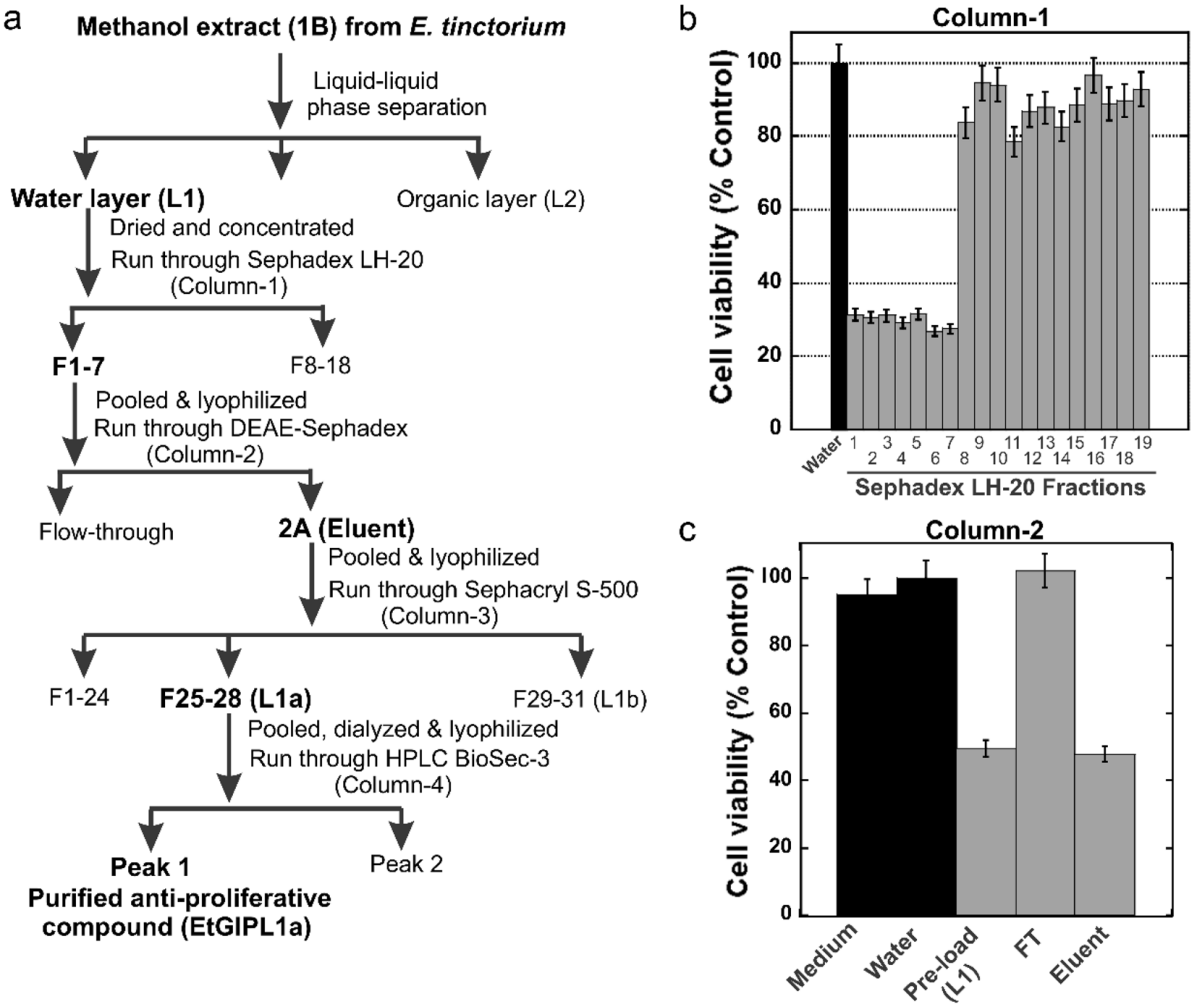
Purification of the anti-proliferative polysaccharide (EtGIPL1a) from *E. tinctorium*. (**a**) Summary of the purification scheme used. (**b**) 1B extract from *E. tinctorium* was purified using Sephadex LH-20 size-exclusion chromatography (column-1). Active fractions (F1-7) were pooled, lyophilized and ran through DEAE-Sephadex anion exchange chromatography (column-2) as shown in (**c**). The eluent (2A) showed anti-proliferative activity while the flow-through (FT) had no activity. Error bars represent S.D. Results shown are representative from three biological replicates.

**Figure 2:**
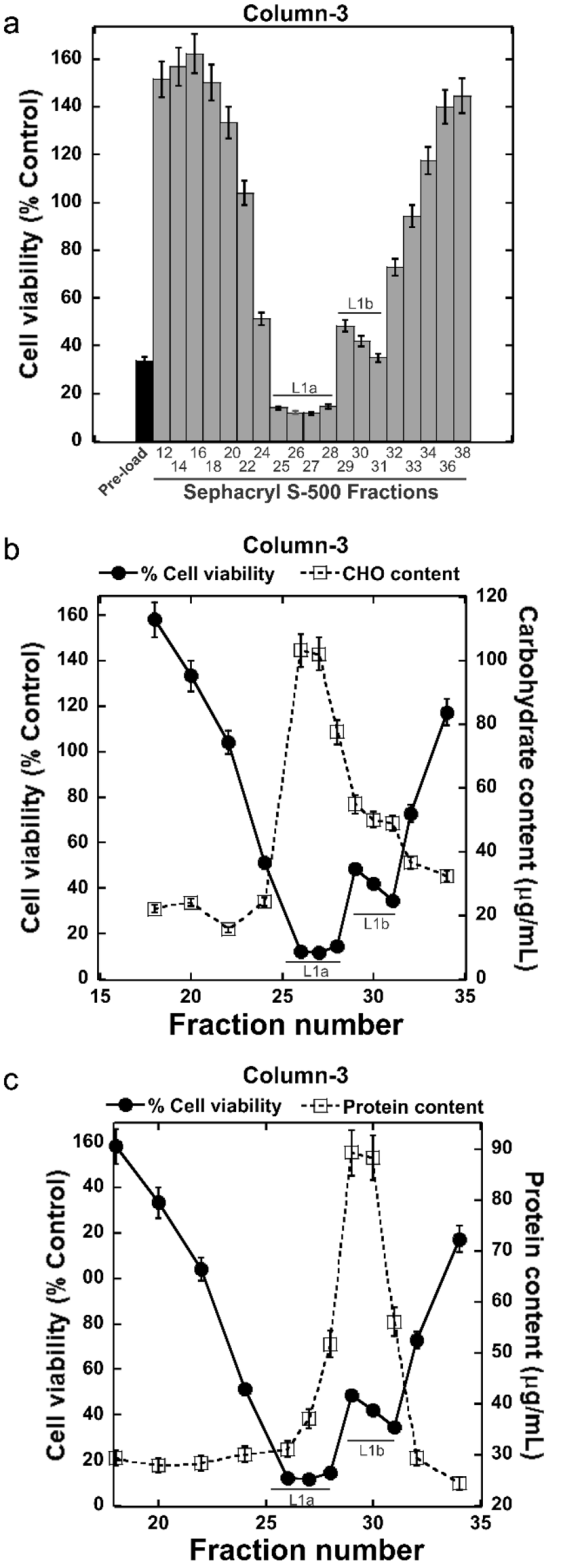
Purification of the anti-proliferative polysaccharide from *E. tinctorium* (EtGIPL1a) using Sephacryl S-500 (column-3). Collected fractions were assessed for cell viability (**a**), carbohydrate (**b**) and protein content (**c**). Error bars represent S.D. and results are representative from three separate experiments.

The overall scheme adopted for the purification of the anti-proliferative compound from *E. tinctorium* (EtGIPL1a), subsequently determined to be a polysaccharide, is shown in Fig. 1a. L1 was first subjected to Sephadex LH-20 size-exclusion chromatography designated as column-1. As shown in Fig. 1b, the major anti-proliferative activity was found in fractions 1–7, suggesting that the anti-proliferative compound had a relatively large molecular weight; these fractions 1–7 from column-1 were then pooled, concentrated, lyophilized, and resuspended in water. It was then subjected to column-2, DEAE-Sephadex anion exchange chromatography using l-Histidine (pH 6.2) as the buffer of choice. The flow-through and eluent from column-2 were concentrated, dialyzed, lyophilized, filter-sterilized, and assessed for anti-proliferative activity. As shown in Fig. 1c, the eluent referred to as 2A (but not the flow-through from DEAE-Sephadex) contained the anti-proliferative activity. This result suggested the presence of acidic groups on the anti-proliferative compound that were effectively associated with positively charged DEAE-Sephadex resin and were eluted upon addition of salt to the mobile phase. Eluent 2A was then subjected to column-3, Sephacryl S-500 high resolution size-exclusion chromatography. As shown in Fig. 2a, the anti-proliferative activity was retained in fractions 25–28 (designated L1a) and 29–31 (designated L1b). The fractions collected from column-3 were also assessed for carbohydrate (CHO) and protein contents. Results in Fig. 2b show that the major anti-proliferative activity L1a correlated strongly with carbohydrate content but not with the protein content. In contrast, the L1b activity appears to correlate better with the protein content (Fig. 2c).

**Figure 3:**
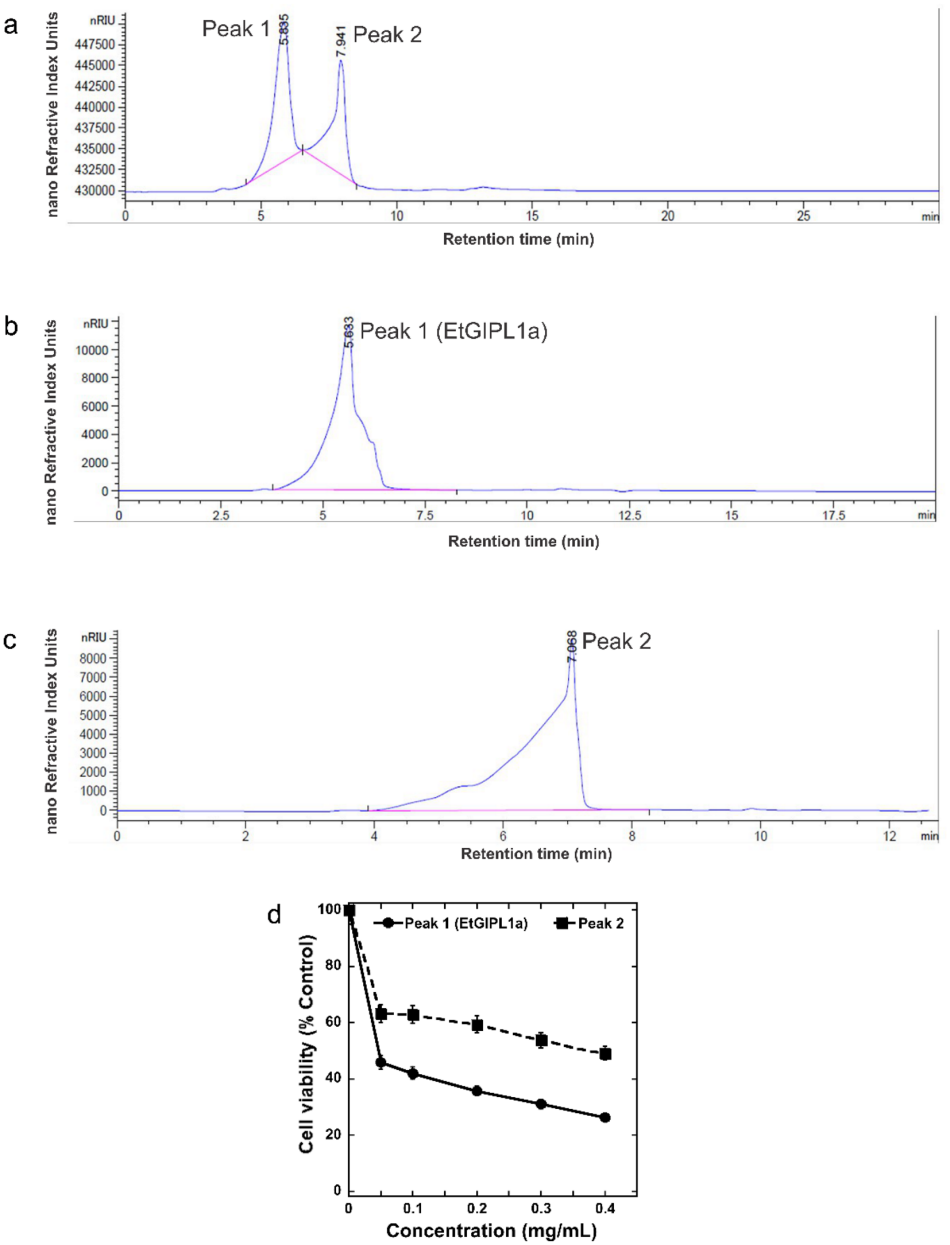
HPLC BioSEC-3 analysis of EtGIPL1a. Full elution profile of (**a**) L1a (**b**) Peak 1 (EtGIPL1a), and (**c**) Peak 2. The collected two peaks in (**b**) and (**c**) were assessed for anti-proliferative activity, as shown in (**d**).

The major bioactive fraction L1a was further purified by HPLC using Agilent BioSEC-3 designated as column-4. As shown in Fig. 3a, the HPLC BioSEC-3 profile of L1a shows two peaks referred to as Peak 1 and Peak 2. These were subjected to further purification by fraction collection. The collected Peak 1 and Peak 2 exhibited a high level of purity (Fig. 3b,c, respectively). Peak 1, designated as EtGIPL1a, has a retention time of 5.633 min while Peak 2 has a retention time of 7.068 min. Figure 3d shows that both EtGIPL1a and Peak 2 exhibited dose-dependent inhibition on proliferation of HeLa cells, with a notably stronger effect by EtGIPL1a. The yield of polysaccharides isolated in the lyophilized sample at each purification step is shown in Supplementary Table S1. Using dextran standards on BioSec-3 column, a standard curve was obtained (Supplementary Fig. S2 online). Based on the standard curve, the peak maxima molecular weight (M_p_) of EtGIPL1a was estimated to be 216,271 g/mol (Da) or 216 kDa (Supplementary Fig. S2 online and Supplementary Table S2 online). We further performed calculations to determine the number (M_n_) and weight average molecular weight (M_w_) of EtGIPL1a. As shown in Supplementary Table S2 online, the M_n_ and M_w_ of EtGIPL1a was calculated to be 232 kDa and 275 kDa, respectively. The dispersity was calculated to be 1.18.

### Fourier transform infrared (FTIR) analysis

FTIR showed the presence of characteristic absorption peaks for a polysaccharide^11^ (Supplementary Fig. S3 online): a broad band at 3283 cm^−1^ that referred to free hydroxyl group stretching and a weak signal at 2923 cm^−1^ for the C–H stretch. There were some other peak stretches as well: 1598 cm^−1^ for carboxyl C=O, 1383 cm^−1^ for C–H bending and 1039 cm^−1^ corresponding to pyranose ring stretching vibration. The observed functional groups present in EtGIPL1a are consistent with that of a typical polysaccharide due to the presence of free O–H groups, pyranose ring, and alkanes^12–14^.

### Monosaccharide composition analysis

The monosaccharide analysis by trimethylsilyl (TMS) methyl glycoside derivatization using GC–MS revealed that EtGIPL1a consisted of predominantly glucose (Glc) (54.3%). Relatively large amounts of galactose (Gal) (19.6%), mannose (Man) (11.1%), and fucose (Fuc) (10.3%) were also found in EtGIPL1a (Supplementary Fig. S4 and Table S3 online). EtGIPL1a also contained glucuronic acid (GlcA) (4.0%) and traces of rhamnose (Rha) (0.6%). For comparison, an immuno-stimulatory polysaccharide EtISPFa, also isolated from *E. tinctorium*, contains significantly higher amount of glucuronic acid (10.1%) and lower amount of fucose (1.8%) (Supplementary Table S3 online)^5^.

### Glycosyl linkage analysis

Consistent with the composition analysis, the glycosyl linkage analysis showed a majority of glucose linkages (%) in EtGIPL1a (Supplementary Fig. S5 online and Table 1). The main glucose linkages were 3-substituted, 6-substituted, 3,6-disubstituted, and terminal. A small amount of 4-substituted glucose was also present. Galactose was found in 6- and 2,6- linkages, and GlcA was 4-substituted. Besides these, there were small amounts of terminal mannose (1.3%) as well as 3-substituted (2.5%) and terminal (0.9%) fucose. Compared with the polysaccharide EtISPFa reported previously^5^, 3-Fuc*p* and 6-Gal*p* were elevated, and 2,6-Gal*p* was found, which had not been detected at all in the EtISPFa polysaccharide.

**Table 1:** Glycosyl linkage analysis of EtGIPL1a by partially methylated alditol acetates. For clarity, residues found at < 1% were omitted.

PMAA	Linkage	Peak area %
1,5-Di-O-acetyl-1-deuterio-6-deoxy-2,3,4-tri-O-methylgalactitol	t-Fuc*p*	0.9
1,5-Di-O-acetyl-1-deuterio-2,3,4,6-tetra-O-methylmannitol	t-Man*p*	1.3
1,5-Di-O-acetyl-1-deuterio-2,3,4,6-tetra-O-methylglucitol	t-Glc*p*	15.3
1,3,5-Tri-O-acetyl-1-deuterio-6-deoxy-2,4-di-O-methylgalactitol	3-Fuc*p*	2.5
1,3,5-Tri-O-acetyl-1-deuterio-2,4,6-tri-O-methylglucitol	3-Glc*p*	28.9
1,5,6- Tri-O-acetyl-1-deuterio-2,3,4-tri-O-methylmannitol	6-Man*p*	2.4
1,5,6- Tri-O-acetyl-1-deuterio-2,3,4-tri-O-methylglucitol	6-Glc*p*	18.3
1,4,5- Tri-O-acetyl-1-deuterio-2,3,6-tri-O-methylglucitol	4-Glc*p*	1.7
1,4,5- Tri-O-acetyl-1,6,6’-trideuterio-2,3,6-tri-O-methylglucitol	4-Glc*p*A	9.2
1,5,6- Tri-O-acetyl-1-deuterio-2,3,4-tri-O-methylgalactitol	6-Gal*p*	3.9
1,3,5,6- Tetra-O-acetyl-1-deuterio-2,4-di-O-methylglucitol	3,6-Glc*p*	13.0
1,2,5,6- Tetra-O-acetyl-1-deuterio-3,4-di-O-methylgalactitol	2,6-Gal*p*	2.6

The overall linkage analysis of EtGIPL1a showed (1 → 3)-linked Glc, (1 → 6)-linked Glc and (1 → 3)(1 → 6)-linked Glc to be the major monomers, indicating that these components are the main chain of EtGIPL1a structure, in similar fashion as described previously for the EtISPFa polysaccharide^5^.

### Nuclear Magnetic Resonance (NMR) analysis

**Figure 4:**
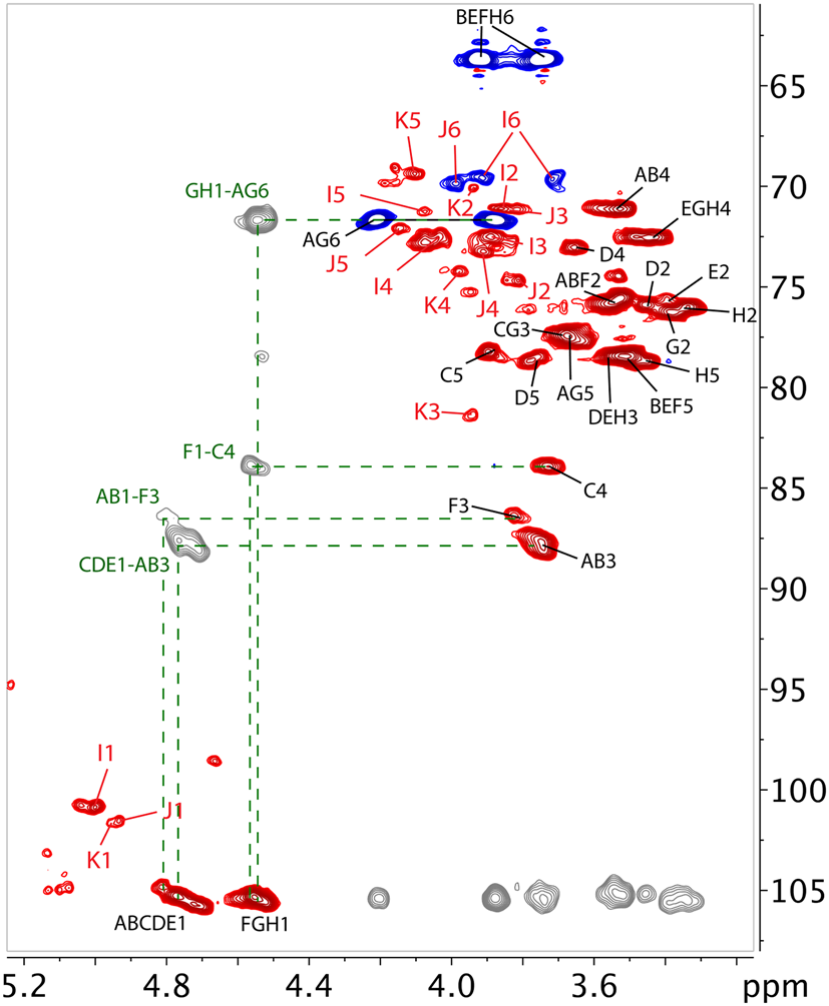
Heteronuclear single quantum coherence (HSQC) and heteronuclear multiple bond correlation (HMBC) NMR analysis of EtGIPL1a. Partial multiplicity-edited ^1^H-^13^C-HSQC NMR spectrum of EtGIPL1a [red (CH groups) and blue (CH_2_ groups) peaks] and partial ^1^H-^13^C-HMBC NMR spectrum (grey peaks, only correlations with anomeric protons/carbons are shown for clarity) of EtGIPL1a. Red labels indicate signals that were of much lower intensity in the EtISPFa sample^5^ (see Table 2), and green lines and labels indicate HMBC correlations.

To gain further structural insights, 1D (Supplementary Fig. S6 online) and 2D NMR analyses were carried out. The spectra were similar to those reported for the EtISPFa polysaccharide^5^, indicating that EtGIPL1a, like EtISPFa, was composed largely of a β-(1 → 3)(1 → 6)-glucan. However, there were several additional signals indicating that the EtGIPL1a polysaccharide was somewhat more complex than the EtISPFa polysaccharide (Fig. 4). We were able to assign three of the additional residues (Table 2), confirming some of the differences observed in the linkage data compared to those of the EtISPFa polysaccharide^5^. We identified 6-substituted α-galactopyranosyl, 2,6-disubstituted α-galactopyranosyl, and 3-substituted α-fucopyranosyl residues A, B, and C, which were found in lower abundance in the EtISPFa polysaccharide than in EtGIPL1a. The anomeric signals of residues A and B showed NOE contacts only with their own H2 and one H6 (Fig. 4 and Table 2). Unlike the H1-H2 correlation, the cross peak between H1 and H6 could only arise from an inter-residue contact, suggesting that both 6-Gal and 2,6-Gal may constitute the backbone of a separate galactan polysaccharide. No NOE signals were detected from H1 of residue C and, therefore, it is unknown whether this residue belonged to the glucan or the galactan. Although it was difficult to quantify the different residues in the NMR experiments because of extensive peak overlap, the intensities of the signals belonging to 4-substituted glucuronic acids were somewhat smaller than in EtISPFa^5^, corroborating the linkage data.

**Table 2:** NMR chemical shift assignments for the residues found in EtGIPL1a. The residues that were not found in EtISPFa^5^ are I, J and K.

No	Residue	Chemical shift (ppm)					
		1	2	3	4	5	6
A	3,6-β-Glc-1 → 3	4.80	3.53	3.79	3.53	3.65	4.20/3.87
		105.1	75.6	87.7	71.2	77.7	71.7
B	3-β-Glc-1 → 3	4.80	3.53	3.74	3.53	3.51	3.91/3.74
		105.1	75.6	87.7	71.2	78.5	63.7
C	4-β-GlcA-1 → 3	4.78	3.46	3.68	3.71	3.90	–
		105.3	75.8	77.5	83.9	78.2	177.5
D	β-GlcA-1 → 3	4.75	3.44	3.54	3.64	3.75	–
		105.5	75.9	78.5	73.1	78.7	177.8
E	β-Glc-1 → 3	4.72	3.36	3.54	3.39	3.49	3.91/3.74
		105.8	76.0	78.6	72.5	78.7	63.7
F	3-β-Glc-1 → 4	4.54	3.54	3.80	3.63	3.51	3.91/3.74
		105.3	75.8	86.4	72.3	78.5	63.7
G	6-β-Glc-1 → 6	4.52	3.38	3.63	3.45	3.63	4.20/3.87
		105.5	76.4	77.4	72.6	77.7	71.7
H	β-Glc-1 → 6	4.51	3.34	3.48	3.42	3.45	3.91/3.74
		105.6	76.0	78.5	72.6	78.7	63.7
I	6-α-Gal*p*-	5.00	3.87	3.89	4.03	4.07	3.92/3.71
		101.0	71.2	72.6	72.8	71.3	69.6
J	2,6-α-Gal*p*-	4.94	3.81	3.86	3.91	4.14	3.99/3.71
		101.4	74.6	71.1	73.2	72.1	69.8
K	3-α-Fuc*p*-	4.95	3.94	3.95	3.96	4.11	1.25
		101.6	70.2	81.3	75.3	69.5	18.6

### Anti-proliferative activity of EtGIPL1a

To determine whether EtGIPL1a has a broader anti-proliferative effect, we assessed it at various concentrations against a panel of human cancer cell lines. Results from the dose-dependent experiments summarized in Table 3 show that EtGIPL1a indeed exhibited strong anti-proliferative effects on the panel of human cancer cell lines. The purified polysaccharide was most effective against DU145 prostate cancer (IC_50_ = 50.6 nM), HCT116 colon cancer (IC_50_ = 122.2 nM) and the two glioblastoma cancer cells, U87 (IC_50_ = 136 nM) and U251 (IC_50_ = 193.2 nM). It was also effective against HeLa cervical cancer (IC_50_ = 287.9 nM), Panc-1 pancreatic cancer (IC_50_ = 343.2 nM), MD-MB-231 breast cancer (IC_50_ = 514.3 nM), SKOV3 ovarian cancer (IC_50_ = 634.2 nM), and MCF-7 breast cancer cells (IC_50_ = 839.5 nM). EtGIPL1a is anti-proliferative on SW480 human colorectal cancer cells with an IC_50_ of 1446 nM, which is tenfold lower than on HCT116 colon cancer cells. We also assessed the effect of EtGIPL1a on SVG immortalized human fetal glial cells^15,16^ commonly used as a comparison with human glioblastoma cell lines such as U251 and U87. The IC_50_ on SVG cells was 144.6 nM (Table 3) which is close to that of U251 and U87, suggesting that EtGIPL1a has no selective activity against human glioblastoma cells.

**Table 3:** IC_50_ of EtGIPL1a against human cancer cell lines. MTT assays were performed 48 h after treatment with EtGIPL1a. The IC_50_ the data shown is an average taken from two independent experiments.

Cell lines	Types	IC_50_ (nM)
DU145	Human prostate cancer	50.6
HCT116	Human colon cancer	122.2
U87	Human glioblastoma	136
SVG	Immortalized human fetal glial cells	144.6
U251	Human glioblastoma	193.2
HeLa	Human cervical cancer	287.9
Panc-1	Human pancreatic cancer	343.2
MD-MB-231	Human breast cancer	514.3
SKOV-3	Human ovarian cancer	634.2
MCF-7	Human breast cancer	839.5
SW480	Human colon cancer	1446

### Mechanism of action of EtGIPL1a

**Figure 5:**
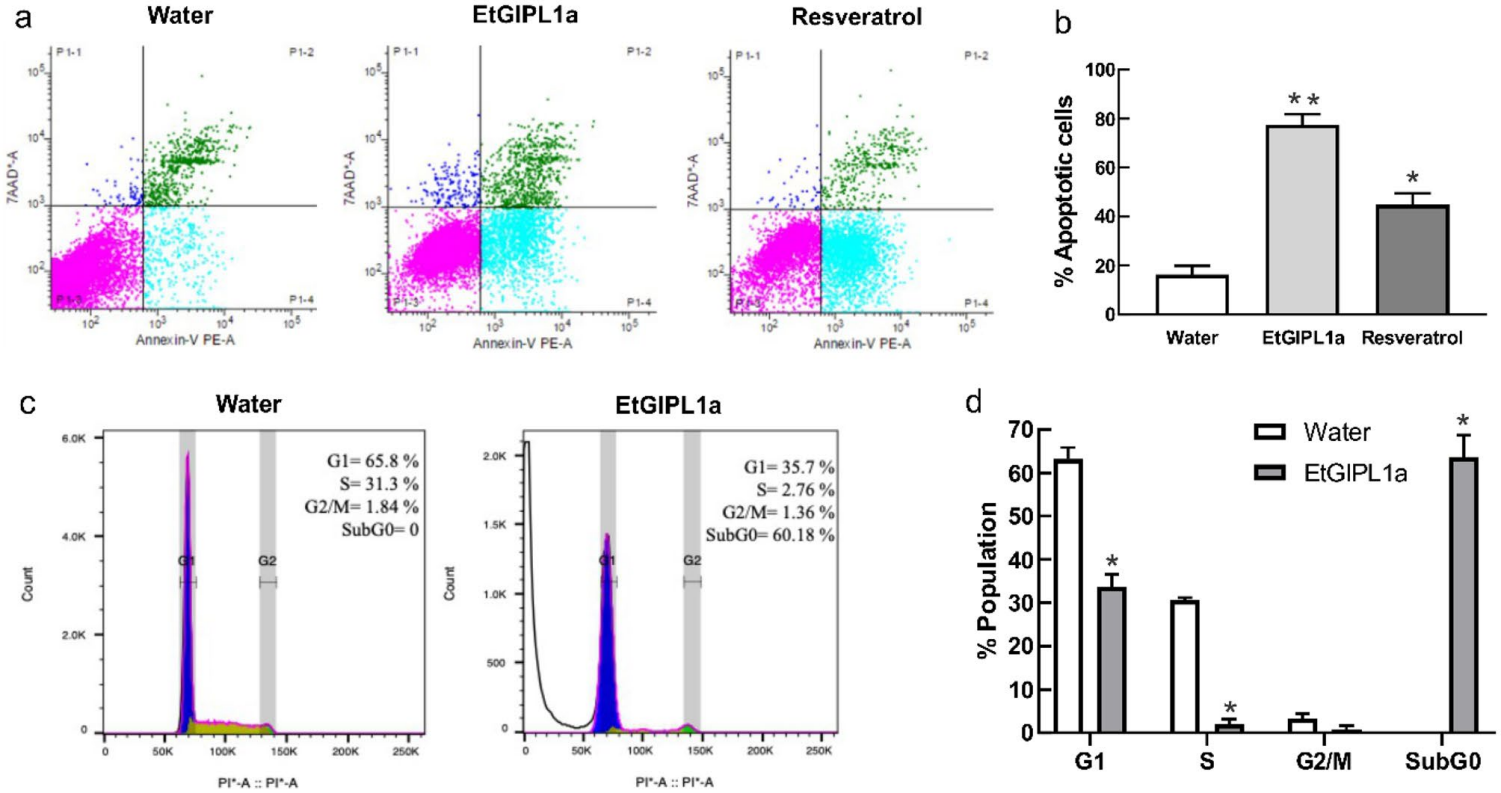
EtGIPL1a induces apoptosis in U251 glioblastoma cells. U251 cells were treated with 27 nM EtGIPL1a, 40 µM resveratrol or water for 48 h. Cell lysates were then subjected to flow cytometry analysis to measure the number of apoptotic cells as shown in (**a**). (**b**) Results from (**a**) and two additional experiments (n = 3) were pooled and plotted as shown. One-way ANOVA was used for statistical analysis. ** shows *p* = 0.0011 and * is *p* = 0.0102. (**c**) A representative result showing the % cell population in G1, S, G2/M and subG0 phases of U251 cell cycle upon treatment with 27 nM EtGIPL1a. (**d**) Results from three biological replicates were pooled and plotted as shown. One-way ANOVA was used for statistical analysis. * indicates p < 0.0001.

To shed light on the mechanism whereby EtGIPL1a exerts its anti-proliferative effect, we first assessed its ability to induce apoptosis. U251 human glioblastoma cell line was chosen for further study because EtGIPL1a has a strong anti-proliferative activity on it (Table 3). Furthermore, glioblastomas are considered very aggressive brain tumors and relatively few treatment options are available^17^. Flow cytometry analysis was used to assess percentages of apoptotic cells and resveratrol, a polyphenol known to induce apoptosis and to have anti-proliferative effect against human cancer cells^18^, was used as a positive control. As shown in Fig. 5a,b, resveratrol at 40 µM significantly induce apoptosis as compared to the negative control water. Based on the IC50 value obtained above, we first used 193 nM of EtGIPL1a to assess its effect on U251 cell apoptosis. Unfortunately, high proportion of cell death was noted leading to insufficient cell materials required for further analysis. At a low concentration of 27 nM, we documented EtGIPL1a to cause an even higher number (80%) of apoptotic U251 cells after treatment for 48 h (Fig. 5b) as compared to resveratrol. The effect of EtGIPL1a on U251 cell cycle was next investigated, also using flow cytometry. As shown in Fig. 5c,d, treatment of U251 cells with 27 nM EtGIPL1a resulted in significant number of cells arrested at the subG0 stage, indicative of cell death. This is consistent with the earlier results that the anti-proliferative polysaccharide induced apoptosis (Fig. 5a,b). We made an interesting preliminary observation on the morphology of U251 cells upon treatment with EtGIPL1a. As shown in the Supplementary Fig. S7a online, cells treated with 14 nM or 27 nM EtGIPL1a displayed stellate shape by Day 2 and by Day 5, more cells appear to display such phenotype. This was not observed in cells treated with the control water (Supplementary Fig. S7a online) nor with yeast β–glucan (Supplementary Fig. S7b online). Such changes in cell morphology suggested that EtGIPL1a could induce U251 cell differentiation. Finally, we assessed proliferation of U251 cells over a 9-day period upon treatment with EtGIPL1a. As shown in Supplementary Fig. S8a online, 14 nM or 27 nM EtGIPL1a inhibited U251 cell proliferation beginning at Day 4 as compared to the control water. In contrast, at similar concentration, the yeast β–glucan stimulated growth of U251 cells as compared to its control, 0.1% DMSO (Supplementary Fig. S8b online).

## Discussion

In this study, we describe the isolation of an anti-proliferative polysaccharide EtGIPL1a with molecular weight of ~ 275 kDa from methanol extract of the North American fungus *E. tinctorium*. EtGIPL1a was isolated from the methanol extract by chloroform-water extraction, Sephadex LH-20, DEAE-Sephadex, Sephacryl S-100 and HPLC BioSEC-3 chromatography. Its size and chemical analyses that include monosaccharide composition, linkage analysis, FTIR, 1D and 2D NMR, suggested that EtGIPL1a is a novel anti-proliferative polysaccharide.

A 1354 kDa immuno-stimulatory polysaccharide from the water extract (EtISPFa) and a 5 kDa anti-inflammatory polysaccharide from the sodium hydroxide extract had recently been isolated from *E. tinctorium*^4,5^. The following evidence suggests that EtGIPL1a could be a sub-fragment of EtISPFa: (1) both have similar (1 → 3)-linked Glc, (1 → 6)-linked Glc and (1 → 3)(1 → 6)-linked Glc main chains; and (2) both are antiproliferative although EtISPFa was more potent against U251 and HeLa cells with IC50 of 13.7 nM and 18.4 nM, respectively. However, we postulate that EtGIPL1a is a polysaccharide distinct from EtISPFa. Firstly, EtGIPL1a was isolated from the 80% ethanol extract while EtISPFa was isolated from the water extract. Secondly, there are significant differences in the composition and abundance of some monosaccharides in both polysaccharides (Table 1, Supplementary Fig. S4 online, Supplementary Table S3 online, and Supplementary Fig. S5 online). EtGIPL1a contains significantly higher amount of fucose (10.3%), mannose (11.1%) and galactose (19.6%), and significantly less glucuronic acid (4%) and rhamnose (0.6%) (Supplementary Table S3 online) compared to EtISPFa, which is corroborated by GC–MS analysis (Table 1). In addition, EtGIPL1a has an undetectable level of xylose and arabinose. Thirdly, EtGIPL1a has 2,6-Galp (2.6%) which was not detected at all in EtISPFa. Finally, EtGIPL1a only has very weak to no immunomodulatory activity (Supplementary Fig. S9) while EtISPFa has immunomodulatory activity which is stronger than the positive control lipopolysaccharide^5^.

To our knowledge, *E. tinctorium* is currently the only fungus shown to contain three distinct polysaccharides with three distinct bioactivities. Most polysaccharides isolated from the same fungus exhibit similar bioactivity. For example, PSCK2-2 and PSCK2-3 from *Cordyceps kyushuensis* have different sugar composition but have similar hydroxy radical scavenging activity^19^. LBPs-5 and LBPs-6 from *Lenzites betulina* with slightly different monosaccharide content showed similar anti-oxidant activity^20^. Six polysaccharides from *Trichoderma harzianum* with differences in their sugar composition all showed anti-oxidant, cytotoxic and enzyme inhibition properties^21^.

EtGIPL1a is an acidic polysaccharide with considerable amount of glucuronic acid (4%), uncommon in fungal polysaccharides. High content of glucuronic acid (10%) was also found in EtISPFa, another bioactive polysaccharide isolated from *E. tinctorium*^5^. Other fungi that contain acidic polysaccharides include *Pleurotus abalonus*, *Fusarium* and *Gibberella* species, as well as *Plectosphaerella cucumerina*, *Verticillium dahliae* and *V. albo-atrum*^22–24^. β-Glucans isolated from *Grifola frondosa*^25^ and *Schizophyllum commune*^26^ share the same structural features as EtGIPL1a and EtISPF1, as their main chain is composed of (1 → 3)-linked Glc with branching at (1 → 6)-linked Glc. According to several studies conducted on fungal polysaccharides, β-1 → 3 linkage in the major backbone with β-1 → 6 branching points is required for the antiproliferative activity^27^.

Polysaccharide composition is known to link with bioactivity. Glucose, mannose, and galactose are amongst the most commonly studied monosaccharide components of mushrooms, whereas glucuronic acid, galacturonic acid, fructose, *N*-acetylglucosamine, *N*-acetylgalactosamine, and ribose are the least studied^14^. According to one study, fucose, glucose and mannose were considered essential for the anti-proliferative properties of two fungi, *Antrodia xantha* and *Rigidoporus ulmarius*; the bioactivity of these fungi was compared with the inactive *A. cinnamomea* and *A. malicola*, which lacked the aforementioned monosaccharides in their structure^28^. Another study showed the anti-proliferative effect of a fucose-containing highly branched 1,3-β-mannoglucan isolated from the fungus *Wolfiporia cocos*, a well-known Chinese medicine. This compound was found to inhibit lung cancer by down-regulating the TGFβR signaling pathway, leading to inhibition of the migration of human metastatic lung cancer cells CL1-5^29^. Another anti-proliferative heterogenous polysaccharide (GIPinv), recently isolated from *Paxillus involutus,* also contained fucose as terminal sugar^30^. A heterogenous high molecular weight fucose-rich polysaccharide fraction called FMS with fucose attached at the terminals was also isolated from *Ganoderma lucidum*^29^. FMS was found to induce production of IgM antibodies against tumor-specific glycans in Lewis lung cancer cells. FMS mediated antibody response and suppressed monocyte chemoattractant protein-1, an inflammatory mediator associated with cancer. Moreover, FMS suppressed Globo H, a carbohydrate antigen only found on the surface of cancer cells. This immunogenic ability of FMS was believed to be due to the presence of terminal fucose in its structure which is capable of interacting with the surface antigens on tumor cells^31^. Further experiments will be required to determine the role of fucose in EtGIPL1a’s anti-proliferative activity and whether it exhibits in vivo properties similar to FMS.

In this study, using U251 glioblastoma cells as a representative, we show that induction of apoptosis is at least one mechanism whereby EtGIPL1a exerts its anti-proliferative effects. This was further corroborated by cell cycle analysis where the polysaccharide was found to predominantly arrest cells at subG0 stage. Interestingly, our preliminary data show that at low concentrations of 27 nM and 14 nM, EtGIPL1a stimulated U251 cells to adopt a stellate-like shape resembling an astrocyte, suggesting that EtGIPL1a could be inducing differentiation in U251 cells. Such morphology was not observed in U251 cells treated with water or yeast β–glucan. In addition, unlike water-, DMSO- or yeast β-glucan-treated cells, EtGIPL1a inhibited U251 cell proliferation for up to 9 days. Differentiation therapy has been shown to be clinically beneficial in the treatment of some hematologic malignancies^32^ and has been proposed as potential approach to treat glioblastoma multiforme^33–35^. Therefore, it will be important to further explore whether EtGIPL1a can truly induce differentiation in cancer cells. For instance, one could investigate whether EtGIPL1a can induce U251 cell differentiation by examining its effect on the expression of glial fibrillary acidic protein, a specific marker for astrocytes.

In conclusion, this is the first study to describe the isolation and characterization of an anti-proliferative polysaccharide from *Echinodontium tinctorium*, a fungus unique to western North America. EtGIPL1a is a complex and unique *β*-glucan polysaccharide rich in galactose, mannose, fucose and glucuronic acid. EtGIPL1a is made up mostly of a backbone consisting of a *β*-(1 → 3)(1 → 6)-glucan containing *β*-(1 → 4)-Glc*p*A residues, along with an α-(1 → 6)-galactan. The polysaccharide is anti-proliferative against 10 tested cancer cell lines, and induction of apoptosis is at least one mode of its anti-proliferative effect. This study has laid the foundation for the further development of EtGIPL1a as a potential medicinal polysaccharide. Future studies should include assessment of its anti-cancer activity in animals, especially its potential use in differentiation cancer therapy. Overall, this and previous studies strongly support that *E. tinctorium* should be considered as a medicinal fungus.

## Methods and materials

### Materials, reagents and cell lines

Eagle’s Minimal Essential Medium was purchased from LONZA (Walkersville, Maryland, USA). 3-(4,5-dimethylthiazol-2-yl)-2-5 diphenyltetrazolium bromide (MTT), dimethylsulfoxide (DMSO) and the dextran standards (T1, T5, T12, T25, T50, T80, T150, T270, T410) were obtained from Sigma-Aldrich (St. Louis, MO, USA). Fetal bovine serum (FBS) was from Life Technologies Inc. (Waltham, Massachusetts, USA). Sephadex LH-20 resin, DEAE-Sephadex, and HiPrep 26/60 Sephacryl S-500 HR pre-packed column were purchased from Cytiva (Marlborough, MA, USA). HPLC BioSEC-3 column and guard column were purchased from Agilent (Santa Clara, CA, USA). Immortalized human fetal glial cell line SVG was a gift from Dr. Eugene O. Major, NINDS, National Institute of Health, Bethesda, Maryland, USA^15,16^. All cell lines were from the American Type Culture Collection. All cells were maintained in Eagle’s Minimal Essential Medium except the following: Panc-1 was maintained in Dulbecco’s Modified Eagle Medium (LONZA). Yeast β–glucan was from Neogen (Lansing, Michigan, USA).

### Collection and extraction of the mushroom

*Echinodontium tinctorium* conks were collected from western hemlock trees (*Tsuga heterophylla*) near Terrace (CL103) and Smithers (CL37), BC, Canada, in August 2014 and 2015, respectively. Voucher specimens for these collections are deposited at the University of Northern British Columbia, BC, Canada. The specimens, previously confirmed using morphological and molecular techniques^4^, were dried in a hot air oven (55 °C, 24–48 h), cut into smaller pieces using a saw machine, and ground to fine powder using a hammer mill. Powdered mushrooms (300 g) were sequentially extracted with 80% ethanol (1.5 L, 65 °C, 3 h). The extract was vacuum filtered through Whatman filter paper No. 3 and the filtrate was designated 1A. The residue was further extracted with 50% methanol (1.5 L, 65 °C, 3 h). The methanol extract was filtered and the filtrate was designated 1B. Crude extract 1B was concentrated, lyophilized, and filter sterilized before assessment for anti-proliferative activity.

### Anti-proliferative assay

Anti-proliferative activity of 1B was assessed by % cell viability using the cytotoxic MTT assay. HeLa cells were plated (96 well, 1.5 × 10^4^ cells/well) and, after 22–24 h, plated cells were treated with crude methanol extract (1B) and phase separated with aqueous layer (L1) for 48 h at concentrations ranging from 0.1 to 1 mg/mL. Cells were observed for morphological changes under a microscope and incubated with 50 μL of MTT solution (3 h, 37 °C). Medium was then removed from the wells and 150 μL DMSO was added and incubated for another 5 min. Different purple color intensities of formazan were observed, indicative of dose-dependent anti-proliferative response. Formazan purple color was quantified by determining the absorbance of samples at 570 nm using Bio-Tek’s Synergy-2 multi-plate reader. The purified polysaccharide EtGIPL1a was assessed against multiple cancer cell lines including HeLa, SW-480, U87, U251, DU145, HCT116, Panc-1, MD-MB-231, MCF-7, and SKOV-3. It was also assessed on SVG immortalized human fetal glial cell line. Cell viability was assessed 48 h after treatment using MTT assay as described above. For time-dependent experiments, U251 cells were treated with EtGIPL1a (14 and 27 nM), yeast β-glucan (10 nM) and controls (water and 1% DMSO) for up to 9 days, with some wells subjected MTT assay on a daily basis.

### Purification of anti-proliferative polysaccharide from *E. tinctorium*

Phase separation was performed by dissolving 500 mg of 1B in water and partitioning with chloroform that resulted in two distinct layers: aqueous (L1) and organic (L2). L1 was then subjected to a 100 mL Sephadex LH-20, designated column-1 (80 mg L1, 1 mL/min, 3 mL fraction size, 2 CV). Collected fractions containing anti-proliferative activity were pooled, lyophilized and subjected to DEAE-Sephadex (designated column-2) using L-Histidine as the running buffer (pH = 5.7–6.4). The column was first equilibrated (l-Histidine buffer, 2 CV, 1 mL/min), followed by sample application (500 mg). Initially, L-Histidine buffer (2 CV) was allowed to run through the column to obtain flow-through (FT). Elution buffer (1 M NaCl in l-Histidine buffer, 2.5 CV) was added and eluent was collected. The FT and eluent were concentrated, dialyzed (MWCO 3500 kDa), lyophilized, filter sterilized and tested for anti-proliferative activity. The bioactive eluent 2A from DEAE-Sephadex was then subjected to Sephacryl S-500 HR SEC designated as column-3. Column-3 was equilibrated (4 CV, 150 mM NaCl, 1.3 mL/min) and injected with 100 mg 2A (2 mL sample loop). Fractions collected (10 mL fraction size, 2.5 CV) were assessed for anti-proliferative activity, carbohydrate (phenol–sulfuric acid method) and protein contents (BCA protein assay, Waltham, MA, USA). The bioactive fractions from column-3 were pooled, dialyzed, lyophilized and designated as L1a.

### Molecular size distribution and purification using HPLC

L1a was subjected to HPLC size-exclusion chromatography for purification and molecular size estimation. The molecular size was estimated by running standard T-series Dextrans. Initially, the HPLC BioSEC-3 column (designated column-4) (Agilent BioSEC-3, 3 µm, 100 A, 7.8 × 300 mm, Guard column Agilent BioSEC-3, 3 µm, 100 A, 7.8 × 50 mm) was equilibrated and then L1a was injected (5–10 μL, 1.2 mL/min, Water) through an autosampler. A Refractive Index Detector (RID) was used for analysis. The HPLC profile for L1a showed two peaks retained at 5.835 and 7.941 min. These peaks were fraction collected (300–400 runs), lyophilized, and assessed for anti-proliferative activity. The bioactive Peak 1 was subsequently named EtGIPL1a.

### Monosaccharide composition analysis

Monosaccharide content of EtGIPL1a was determined by GC–MS as previously described^5^. EtGIPL1a (330 µg) was heated in 1 M methanolic HCl in a sealed screw-top glass test tube for 18 h at 80 °C. After cooling and removal of the solvent under a stream of nitrogen, the sample was treated with a mixture of methanol, pyridine, and acetic anhydride for 30 min to re-*N*-acetylate any amino sugars that might be present. The solvents were evaporated, and the sample was derivatized with Tri-Sil (Pierce) at 80 °C for 30 min. Following extraction with hexane, GC–MS analysis of trimethylsilyl (TMS) methyl glycosides was performed on an Agilent 7890A GC interfaced to a 5975C MSD, using a Supelco Equity-1 fused silica capillary column (30 m × 0.25 mm ID).

### Methylation and linkage analysis

A recently published protocol was adopted^36^. One mg of EtGIPL1a was suspended in 200 µL of DMSO, methylated using dimsyl potassium base, and acetylated using *N*-methylimidazole and acetic anhydride. The samples were extracted with dichloromethane, and the carboxylic acid methyl esters were reduced using lithium aluminum deuteride in THF (80 °C, 8 h). After desalting using an On-guard H^+^ column (ThermoFisher), the samples were remethylated using two rounds of treatment with sodium hydroxide (15 min each) and methyl iodide (45 min each). The samples were then hydrolyzed using 2 M TFA (2 h in sealed tube at 120 °C), reduced with NaBD_4_, and acetylated using acetic anhydride/TFA. The resulting PMAAs were analyzed on an Agilent 7890A GC interfaced to a 5975C MSD (mass selective detector, electron impact ionization mode); separation was performed on a 30 m Supelco SP-2331 bonded phase fused silica capillary column.

### Structural elucidation by spectral analysis

EtGIPL1a was subjected to functional group analysis by FTIR (Bruker ATR-FTIR spectrophotometer by Billerica, MA, USA) with a detection wave range of 4000–400 cm^−1^. Twenty-two scans were obtained, and an IR spectrum was generated using OPUS software. For further structural analysis, NMR was used as previously described^5^. 1D proton NMR (H^1^-NMR) and 2D NMR including ^1^H-^1^H-correlation spectroscopy (COSY), total correlation spectroscopy (TOCSY), nuclear Overhauser effect spectroscopy (NOESY), ^1^H-^13^C-NMR heteronuclear single quantum correlation spectroscopy (HSQC), HSQC-TOCSY, and heteronuclear multiple bond correlation (HMBC) were carried out on an Agilent Inova 600 MHz NMR.

### Apoptosis and cell cycle analyses

Flow cytometry analysis was used to assess effect on apoptosis and cell cycle. U251 cells were plated at 5 × 10^5^ cells/well in 6-well plates and treated with 27 nM of EtGIPL1a or 40 µM Resveratrol for 48 h. Cells treated with water were included as control. Cells were trypsinized followed by centrifugation and washed twice with PBS. For apoptosis analysis, live cells were stained with PE Annexin V and 7-AAD as according to manufacturer’s instructions for Apoptosis Detection Kit I (BD Pharmingen). For cell cycle analysis, the BD Cycletest Plus DNA Reagent Kit (BD Pharmingen) was used as according to the manufacturer’s instructions. Flow cytometry analysis was performed using a BD FACSMelody cell sorter (BD Biosciences) and BD FACS Chorus software (V 1.0). For apoptosis, a total of 10,000 events were recorded for each sample. The percentage of viable and apoptotic cells was calculated from FACS Chorus V1.0. For cell cycle analysis, a total of 40,000 events were recorded for each sample. The percentage of cells residing in each G1, S, G2/M phase were analyzed using FlowJo v10.7.2 Software (BD Life Sciences)^37^.

### Statistical analysis

Most biological experiments were performed three times and with three replicates, and where appropriate, the results were expressed as mean ± SD values of 3 observations. The results were analyzed using one-way analysis of variance (ANOVA) on GraphPad Prism version 8 and *p* < 0.05 was c onsidered significant.

## Supplementary Information

Supplementary Information.

## Data Availability

The datasets used and/or analysed during the current study are available upon request from the corresponding author.

## References

[1] Liu SL, Zhao Y, Dai YC, Nakasone KK, He SH (2017). Phylogeny and taxonomy of *Echinodontium* and related genera. Mycologia.

[2] Ginns, J. H. *Polypores of British Columbia.* Province of British Columbia, Victoria, BC, Tech Rep. **104,** 260 (2017).

[3] Ye Y, Josten I, Arnold N, Steffan B, Steglich W (1996). Isolation of a fluorone pigment from the Indian paint fungus *Echinodontium tinctorium* and *Pyrofomes albomarginatus*. Tetrahedron.

[4] Javed S (2019). Anti-Inflammatory activity of the wild mushroom, *Echinodontium tinctorium*, in RAW264.7 macrophage cells and mouse microcirculation. Molecules.

[5] Zeb M (2021). Structural elucidation and immuno-stimulatory activity of a novel polysaccharide containing glucuronic acid from the fungus *Echinodontium tinctorium*. Carbohydr. Polym..

[6] Bond FT, Fullerton DS, Sciuchetti LA, Catalfomo P (1966). The isolation and structure of echinodol, a triterpene acetate. J. Am. Chem. Soc..

[7] Suzuki S, Murayama T, Shiono Y (2005). Illudalane sesquiterpenoids, echinolactones A and B, from a mycelial culture of *Echinodontium japonicum*. Phytochemistry.

[8] Arnone A, De Gregorio C, Nasini G, Vajna de Pava O (1998). Isolation and structure elucidation of tsugicolines F–H, novel furosesquiterpenes, and tsugicoline I from the fungus *Laurilia tsugicola*. Tetrahedron.

[9] Kanematsu A, Natori S (1972). Triterpenoids of *Echinodontium tsugicola*. Chem. Pharma. Bull..

[10] Smith, A. W. T. *Investigating British Columbia wild mushrooms for growth-inhibitory activity*. MSc. Thesis. The University of Northern British Columbia, Prince George, BC, Canada (2017).

[11] Cui, S. W. Food *Carbohydrates: Chemistry, Physical Properties, and Applications*, 70–72 (ed. Cui, S.) (CRC Press, 2005).

[12] Chen Q (2012). Chemical characterization and immunostimulatory effects of a polysaccharide from *Polygoni Multiflori Radix Praeparata* in cyclophosphamide-induced anemic mice. Carbohydr. Polym..

[13] Liu X (2015). Structure characterization and antitumor activity of a polysaccharide from the alkaline extract of king oyster mushroom. Carbohydr. Polym..

[14] Wang Q, Wang F, Xu Z, Ding Z (2017). Bioactive mushroom polysaccharides: a review on monosaccharide composition, biosynthesis and regulation. Molecules.

[15] Major EO (1985). Establishment of a line of human fetal glial cells that supports JC virus multiplication. Proc. Natl. Acad. Sci. USA.

[16] Ferenczy MW (2013). Clonal immortalized human glial cell lines support varying levels of JC virus infection due to differences in cellular gene expression. J. Neuroimmune Pharmacol..

[17] Van Solinge TS, Nieland L, Chiocca EA, Broekman MLD (2022). Advances in local therapy for glioblastoma—Taking the fight to the tumour. Nat. Rev. Neurol..

[18] Ho Y (2017). Biological mechanisms by which antiproliferative actions of resveratrol are minimized. Nutrients.

[19] Zhang G (2015). Purification and antioxidant effect of novel fungal polysaccharides from the stroma of *Cordyceps kyushuensis*. Industr. Crops Prod..

[20] Guo L (2021). Isolation, structure characteristics and antioxidant activity of two water-soluble polysaccharides from *Lenzites betulina*. BMC Chem..

[21] Saravanakumar K (2021). Isolation of polysaccharides from *Trichoderma harzianum* with antioxidant, anticancer and enzyme inhibition properties. Antioxidants.

[22] Shi X, Zhao Y, Jiao Y, Shi T, Yang X (2013). ROS-dependent mitochondria molecular mechanisms underlying antitumor activity of *Pleurotus abalonus* acidic polysaccharides in human breast cancer MCF-7 cells. PLoS ONE.

[23] Ahrazem O (2000). An acidic water-soluble cell wall polysaccharide: a chemotaxonomic marker for *Fusarium* and *Gibberella*. Mycol. Res..

[24] Ahrazem O (2006). Structural elucidation of fungal polysaccharides isolated from the cell wall of *Plectosphaerella cucumerina* and *Verticillium* spp. Carbohydr. Res..

[25] Fang J (2012). Structure of a β-glucan from *Grifola frondosa* and its antitumor effect by activating Dectin-1/Syk/NF-κB signaling. Glycoconjugate J..

[26] Klaus A (2011). Antioxidative activities and chemical characterization of polysaccharides extracted from the basidiomycete *Schizophyllum commune*. LWT-Food Sci. Tech..

[27] Wasser SP (2002). Medicinal mushrooms as a source of antitumor and immunomodulating polysaccharides. Appl. Microbiol. Biotech..

[28] Chen SC, Lu MK, Cheng JJ, Wang DL (2005). Antiangiogenic activities of polysaccharides isolated from medicinal fungi. FEMS Micro. Letts..

[29] Lin T-Y, Lu M-K, Chang C-C (2020). Structural identification of a fucose-containing 1,3-β-mannoglucan from *Poria cocos* and its anti-lung cancer CL1-5 cells migration via inhibition of TGFβR-mediated signaling. Int. J. Biol. Macromol..

[30] Barad A (2018). Anti-proliferative activity of a purified polysaccharide isolated from the basidiomycete fungus *Paxillus involutus*. Carbohydr. Polym..

[31] Liao SF (2013). Immunization of fucose-containing polysaccharides from Reishi mushroom induces antibodies to tumor-associated Globo H-series epitopes. Proc. Natl. Acad. Sci. USA.

[32] Leszczyniecka M, Roberts T, Dent P, Grant S, Fisher PB (2001). Differentiation therapy of human cancer: Basic science and clinical applications. Pharmacol. Ther..

[33] Xing F (2017). The anti-Warburg effect elicited by the cAMP-PGC1α pathway drives differentiation of glioblastoma cells into astrocytes. Cell Rep..

[34] Caren H, Beck S, Pollard SM (2016). Differentiation therapy for glioblastoma—Too many obstacles?. Mol. Cell. Oncol..

[35] Basile MS (2018). Anticancer and differentiation properties of the nitric oxide derivative of Lopinavir in human glioblastoma cells. Molecules.

[36] Hotchkiss AT (2022). Cranberry arabino-xyloglucan and pectic oligosaccharides induce lactobacillus growth and short-chain fatty acid production. Microorganisms.

[37] FlowJo Software (for Windows) [software application] Version 10.7.2. Ashland, OR: Becton, Dickinson and Company; 2021.

