# Supplementary material for: Isolation and characterization of an anti-proliferative polysaccharide from the North American fungus Echinodontium tinctorium
Source: Sci Rep. 2022 Oct 14;12:17298. doi: 10.1038/s41598-022-21697-0 (PMC9568501; doi:10.1038/s41598-022-21697-0)
Supplement: Supplementary file 1 — Supplementary Information. [file 41598_2022_21697_MOESM1_ESM.doc]

**Isolation and Characterization of an anti-proliferative polysaccharide from the North American fungus *Echinodontium tinctorium***

**Mehreen Zeb^1^, Wai Ming Li^1^, Christian Heiss^3^, Ian Black^3^, Linda E. Tackaberry^2^, Hugues B. Massicotte^2^, Keith N. Egger^2^, Kerry Reimer^1^, Parastoo Azadi^3^, and Chow H. Lee^1*^**

^1^Department of Chemistry and Biochemistry, University of Northern British Columbia, Prince George, BC, V2N 4Z9, Canada.

^2^Department of Ecosystem Science and Management, University of Northern British Columbia, Prince George, BC, V2N 4Z9, Canada.

^3^Complex Carbohydrate Research Center, University of Georgia, Athens, Georgia, 30602, USA.

***Corresponding author:** Department of Chemistry and Biochemistry, University of Northern British Columbia, 3333 University Way, Prince George, BC V2N 4Z9, Canada. Tel.: 250-960-5413; Fax: 250-960-5170; E-mail address: [chow.lee@unbc.ca](mailto:chow.lee@unbc.ca) (Chow H. Lee).

**Supplementary Information**

**Table of Contents**

**Figure S1.** Chemical extraction of *E. tinctorium* and assessment of the ethanol extract 1B for anti-proliferative activity against HeLa cells…………………………………………………………………………….2

**Figure S2.** Estimating the peak maxima molecular weight (Mp) of EtGIPL1a………………………………..2

**Figure S3.** The GC-MS chromatograms from glycosyl composition analysis using TMS derivatization………………………………………………………………………………………………………….3

**Figure S4.** GC-MS chromatogram resulting from glycosyl linkage analysis of neutral and uronic acid residues……………………………………………………………………………………………………………….3

**Figure S5.** FTIR spectrum of EtGIPL1a…………………………………………………………………………..4

**Figure S6.**  ^1^H-NMR spectrum of EtGIPL1a.……………………………………………………………………..4

**Figure S7.** Effect of EtGIPL1a on U251 cell morphology……………………………………………………….5

**Figure S8.** Time-dependent effect of EtGIPL1a and yeast β–glucan on U251 cell viability…………………6

**Figure S9.** Effect of EtGIPL1a on immunostimulatory activity………………………..………………..............7

**Table S1.** Yield of polysaccharides at each purification step…………………………………………………...8

**Table S2.** Calculating the number (M_n_) and weight average molecular weight (M_w_) of EtGIPL1a …………9

**Table S3.** Monosaccharide composition of EtGIPL1a and EtISPFa………………………………………….10

References…………………………………………………………………………………………………………..10


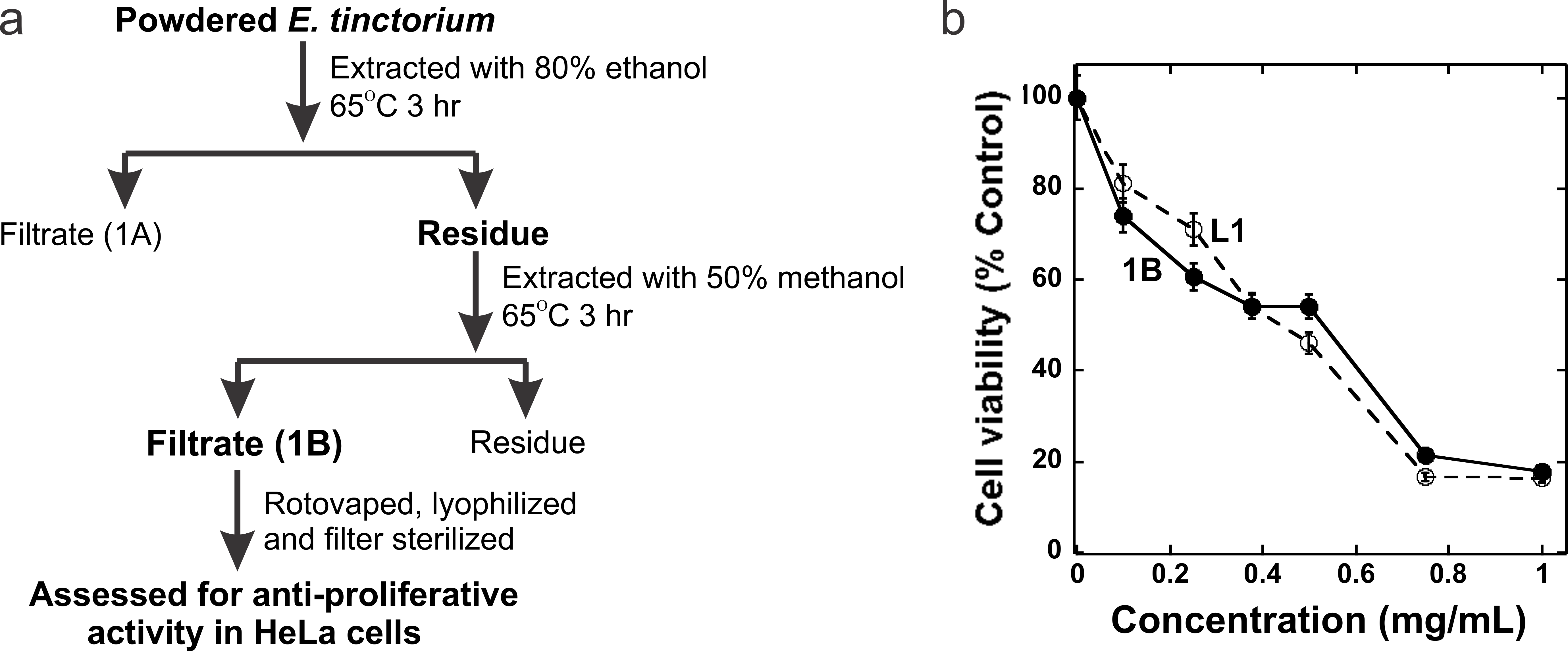


**Figure S1.** Chemical extraction of *E. tinctorium* and assessment of the ethanol extract 1B for anti-proliferative activity against HeLa cells. (**a**) Chemical extraction scheme to obtain methanol extract from *E. tinctorium*. (**b**) Dose-dependent anti-proliferative MTT assay shows inhibition in HeLa cells by 1B methanol extract and L1 from 0.1-1 mg/mL. Error bars represent S.D. Results shown are representative from three biological replicates.


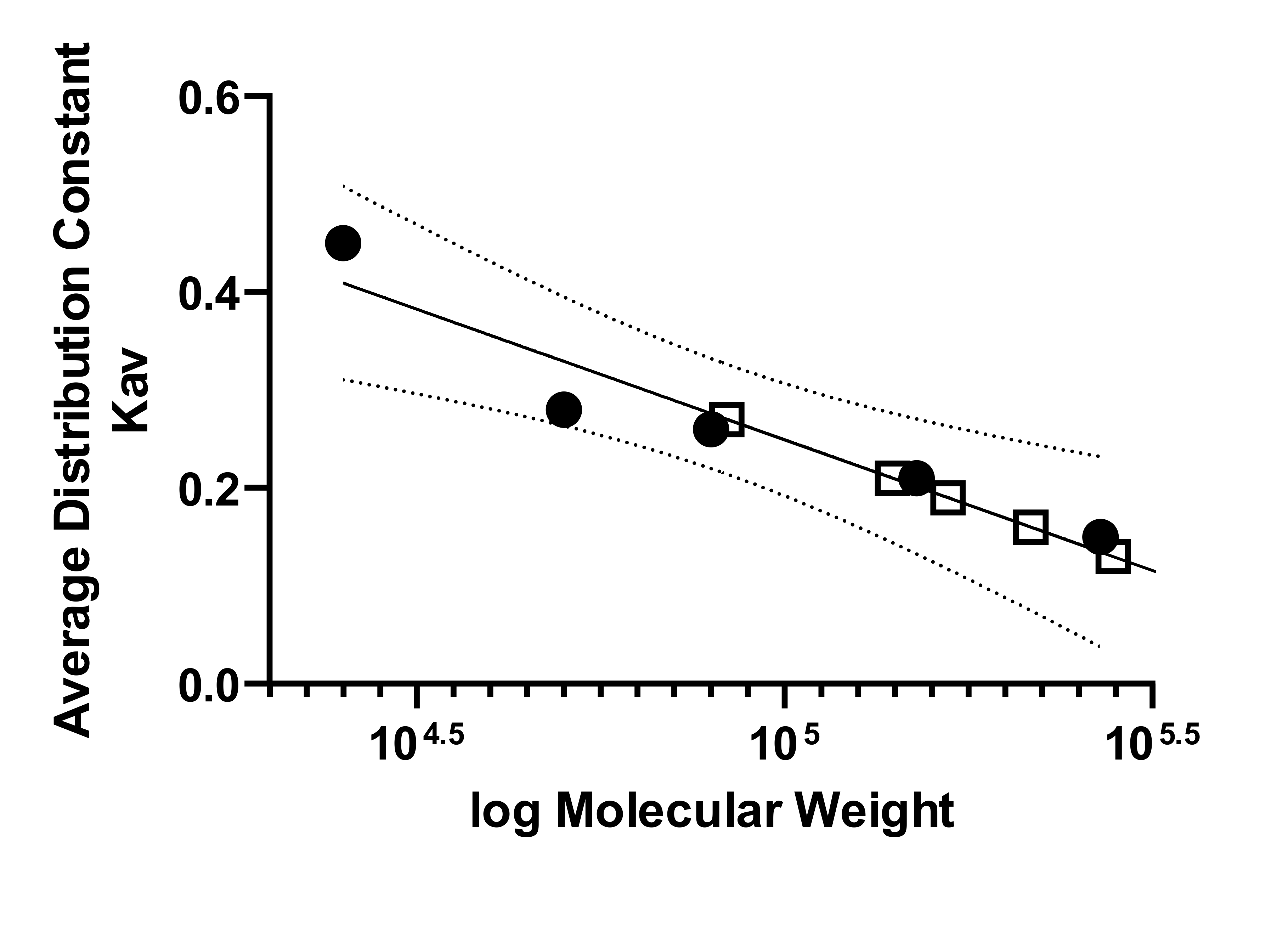


**Figure S2.** Estimating the peak maxima molecular weight (Mp) of EtGIPL1a using HPLC BioSEC-3. Dextran standards and EtGIPL1a were loaded onto BioSEC-3 at a flow rate of 1.2 mL/min. This was used to convert the retention time (Rt) to retention volume, and then to the Average Distribution Constant (Kav) as shown in Table S2. Closed circles represent the Dextran Standards used and open squares are all points of the interpolated molecular weight distribution of EtGIPL1a.


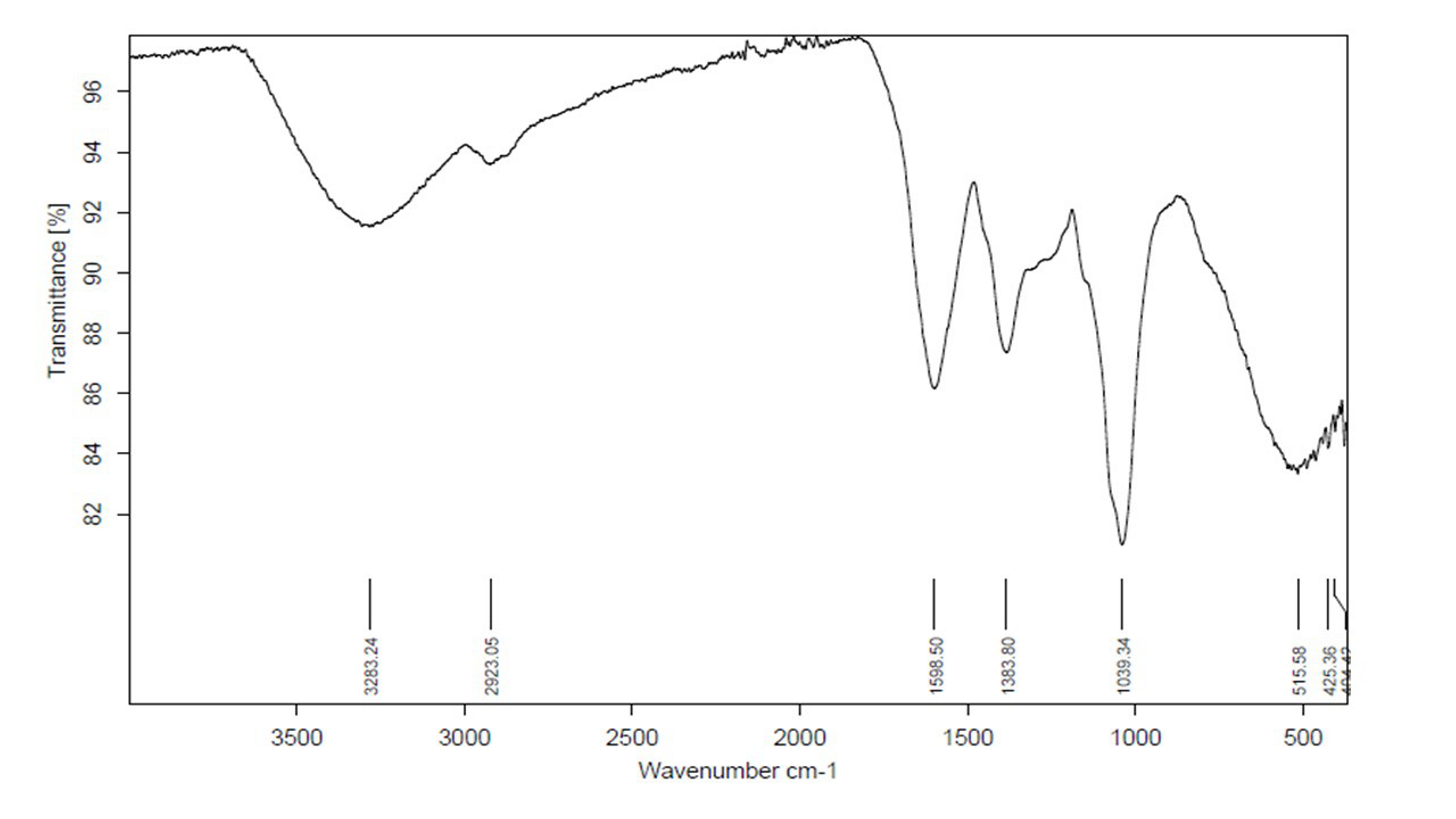
**Figure S3.** FTIR spectrum of EtGIPL1a.

**
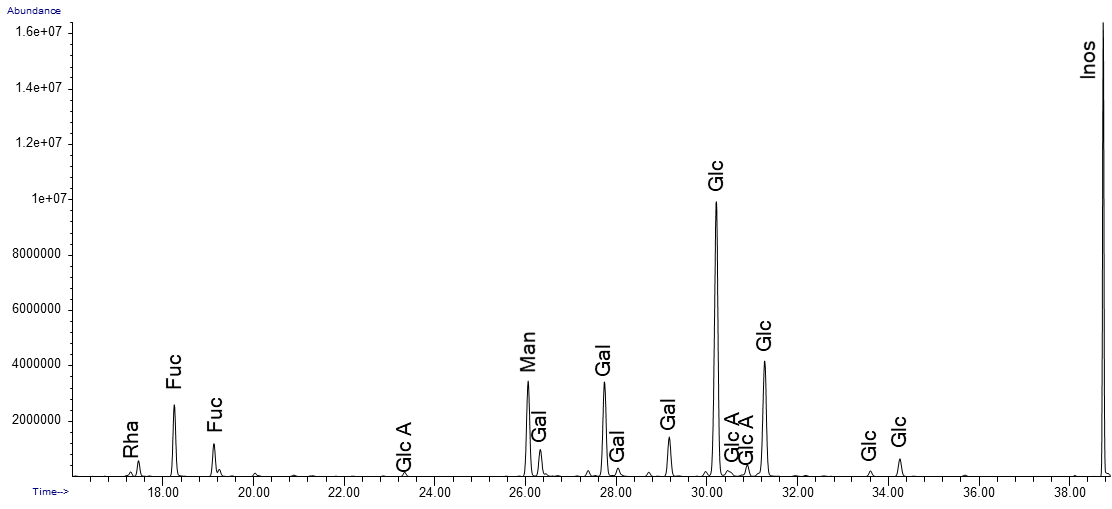
**

**Figure S4.** The GC-MS chromatograms from glycosyl composition analysis using TMS derivatization.

**
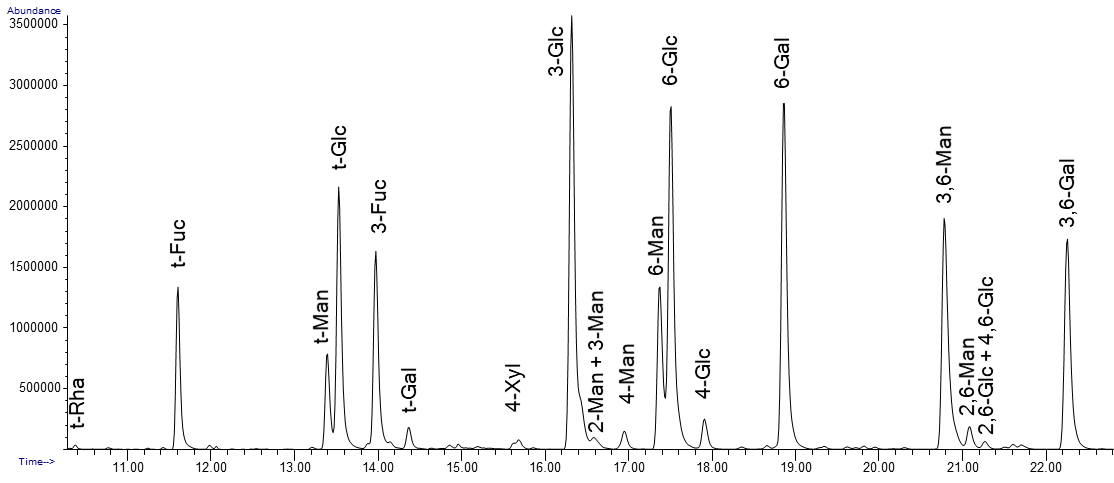
**

**Figure S5.** GC-MS chromatogram resulting from glycosyl linkage analysis of neutral and uronic acid residues. Glycosyl linkage analysis was performed by partially methylated alditol acetates (PMAAs). In the interest of clarity, PMAAs below 1% are not labeled.

**
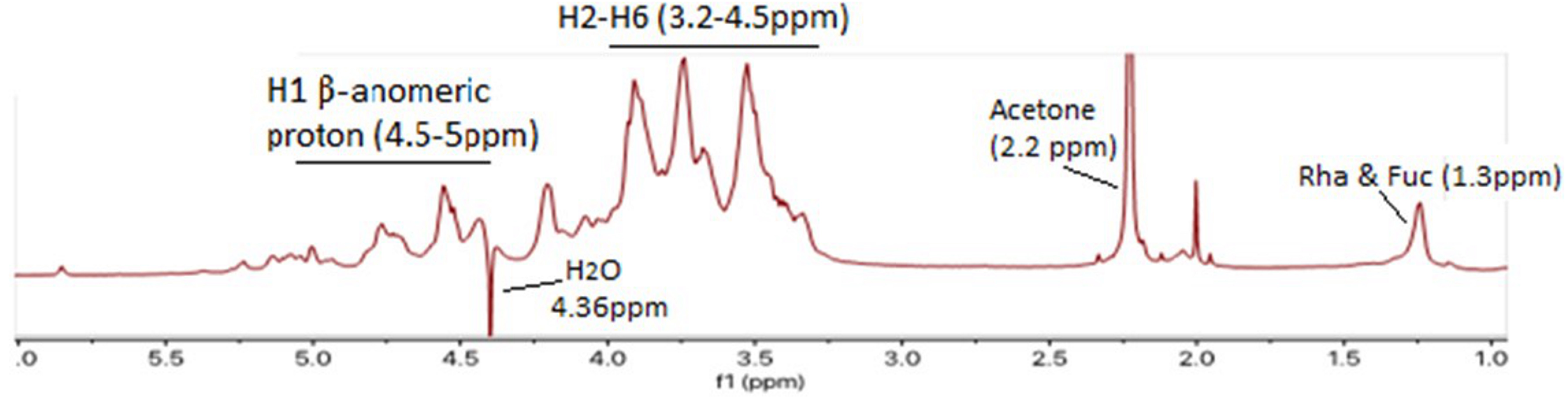
**

**Figure S6.** ^1^H-NMR spectrum of EtGIPL1a.


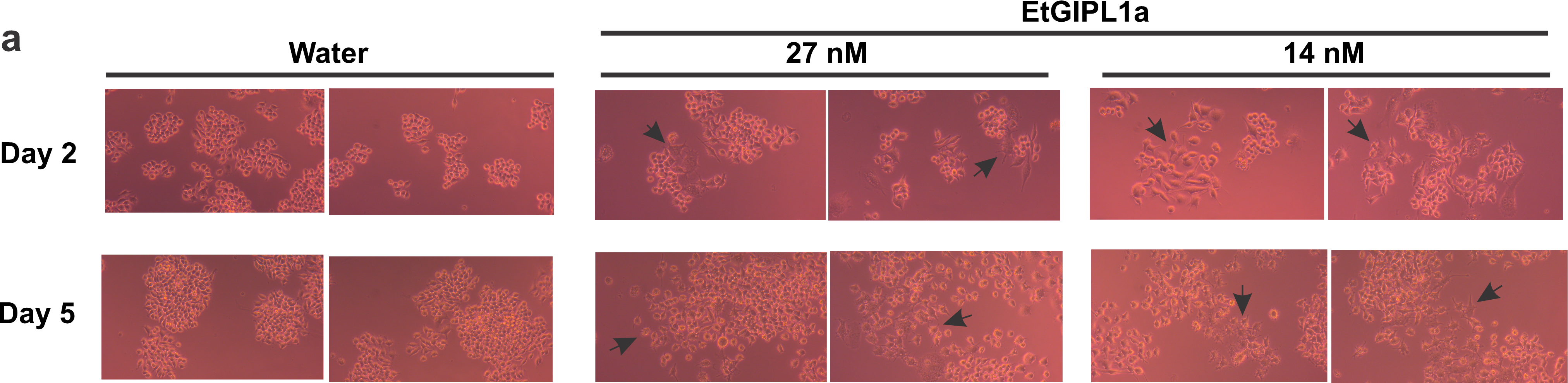


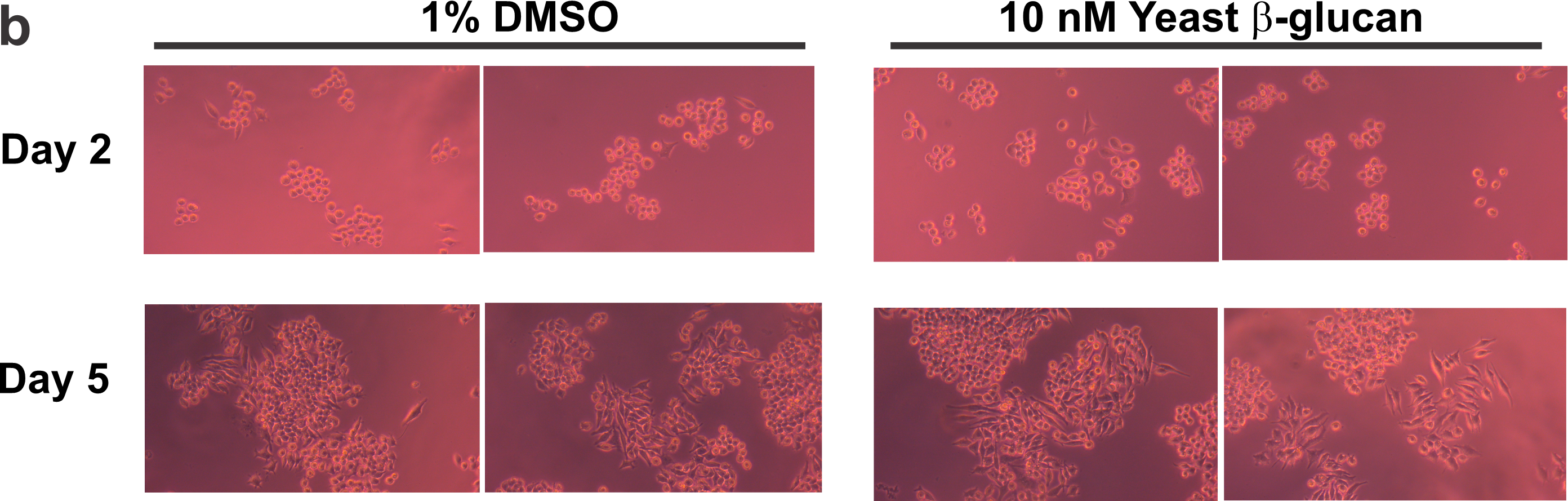


**Figure S7.** Effect of EtGIPL1a on U251 cell morphology. (**a**) U251 cells were incubated with 14 nM or 27 nM EtGIPL1a for 2 days and 5 days as shown. Water (the solvent in which EtGIPL1a was dissolved) was used as a control. (**b**) Similarly, U251 cells were treated with 10 nM yeast β–glucan. 1% DMSO served as the control because it was used to dissolve yeast β–glucan. The two images for each panel were taken from two separate wells. Arrows indicate cells with stellate shape.

**
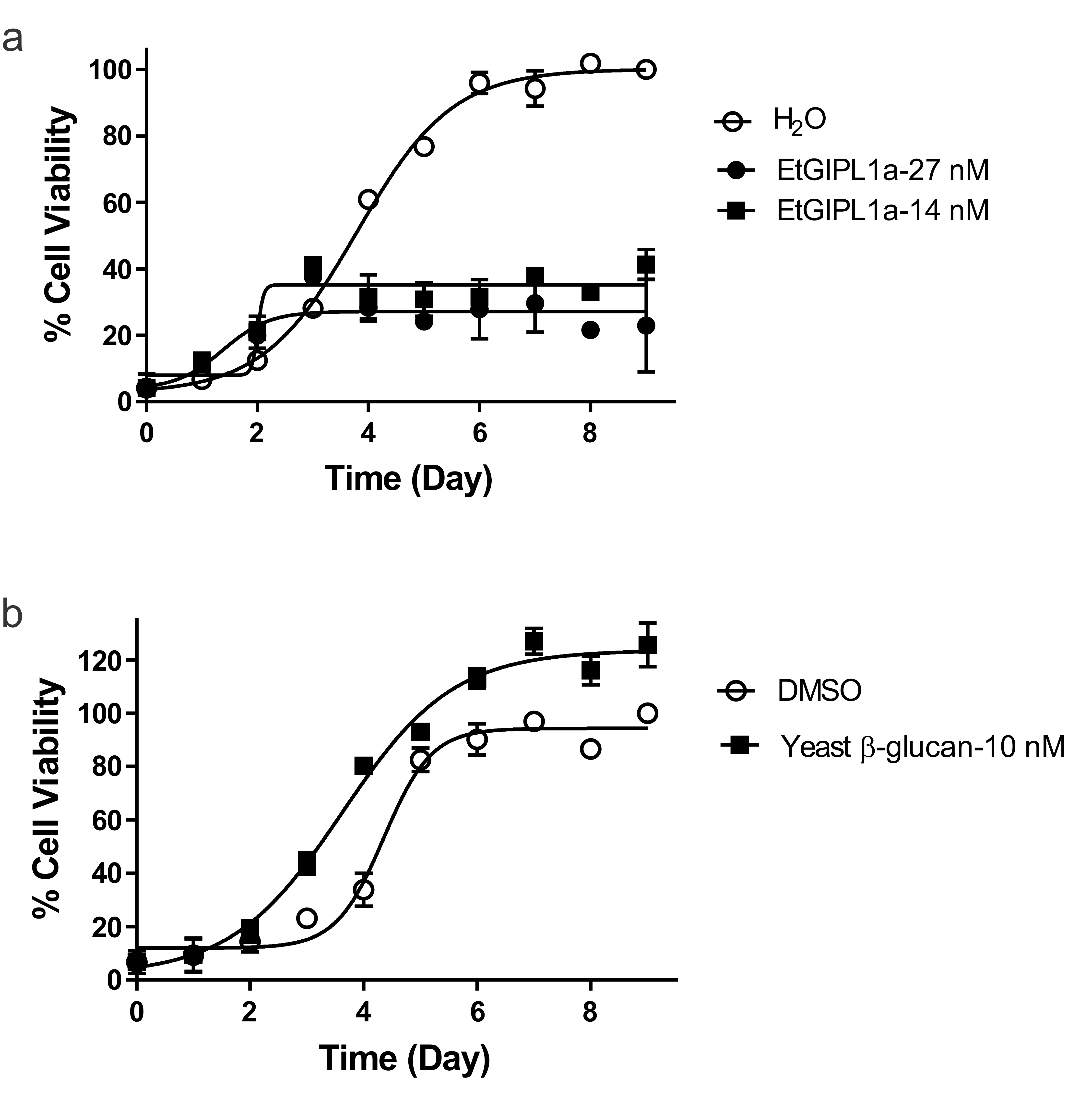
**

**Figure S8.** Time-dependent effect of EtGIPL1a and yeast β–glucan on U251 cell viability. (**a**) U251 cells were treated with 14 nM or 27 nM EtGIPL1a for various times as shown and subjected to MTT assay. Water was used as control. Absorbance values obtained at various time points were expressed relative to the value at Day 0, that is the day when cells were plated. (**b**) U251 cells were treated with 10 nM yeast β–glucan or 1% DMSO which served as the control.


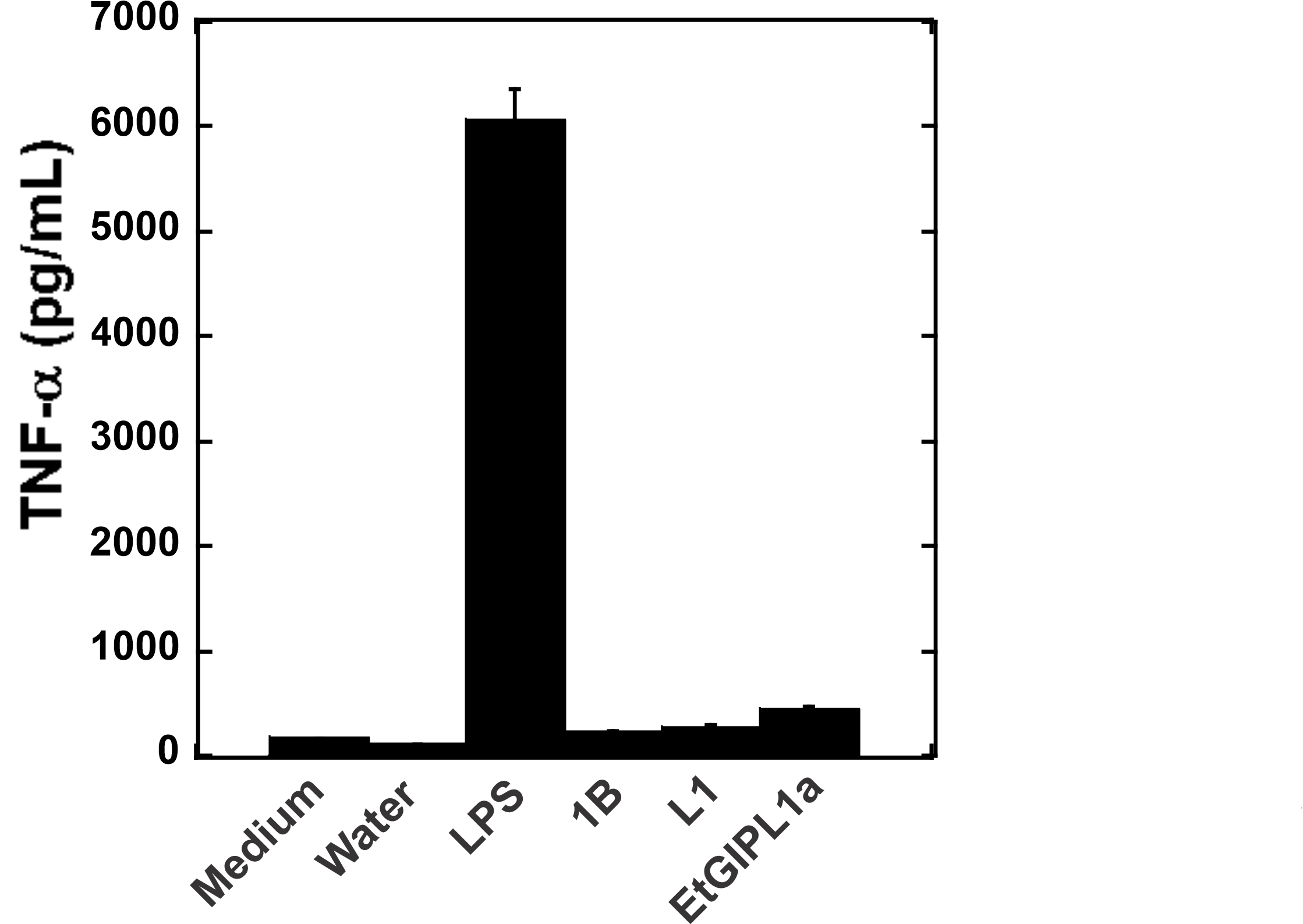


**Figure S9.** Effect of EtGIPL1a on immunostimulatory activity. Raw264.7 macrophage cells were treated with EtGIPL1a (1 mg/mL) and its semi-purified form E2 (1 mg/mL) and L1 (1 mg/mL) for 6 h and the amount of TNF-α production as an indication of immunostimulation was measured as previously described^5^. Data shown is a representative from two independent experiments.

|  | **Preload (mg)** | **Total mass obtained (mg)** | **% Yield** |
| --- | --- | --- | --- |
| *E. tinctorium* powder | 300,000 | 4,250 | 1.4 |
| Methanol extract 1B | 500 | 365 of L1 | 73 |
| Sephadex LH-20 | 80 | 15 | 18.8 |
| DEAE-Sephadex | 500 | 80 of 2A | 16 |
| Sephacryl S-500 | 105 | 11 of L1a | 10.5 |
| HPLC BioSec-3 | 70 | 8 of EtGIPL1a | 11.4 |

**Table S1.** Yield of polysaccharides at each purification step.

| **Rt time (min)** | **Rt volume (mL)** | **Ve-Vo** | **Kav=Ve-Vo/Vc-Vo** | **Mwt (log)** | **MW or Mi (g/mol)** | **Moles** | **ni (number of molecules)** | **niMi** | **wi** | **wiMi** |
| --- | --- | --- | --- | --- | --- | --- | --- | --- | --- | --- |
| 5 | 6 | 0.74 | 0.0814978 | 5.635 | 431519 | 4.63479E-11 | 2.79107E+13 | 1.2044E+19 | 0.191902 | 82809.29 |
| 5.2 | 6.24 | 0.98 | 0.1079295 | 5.522 | 332659 | 6.01216E-11 | 3.62052E+13 | 1.2044E+19 | 0.191902 | 63837.87 |
| 5.4 | 6.48 | 1.22 | 0.1343612 | 5.447 | 279898 | 7.14546E-11 | 4.303E+13 | 1.2044E+19 | 0.191902 | 53712.94 |
| 5.6 | 6.76 | 1.5 | 0.1651982 | 5.335 | 216271^a^ | 9.24766E-11 | 5.56894E+13 | 1.2044E+19 | 0.191902 | 41502.8 |
| 5.8 | 6.96 | 1.7 | 0.1872247 | 5.222 | 166724 | 1.19959E-10 | 4.303E+13 | 7.1741E+18 | 0.114308 | 19057.92 |
| 6 | 7.2 | 1.94 | 0.2136564 | 5.147 | 140281 | 1.42571E-10 | 3.62052E+13 | 5.0789E+18 | 0.080924 | 11352.14 |
| 6.4 | 7.68 | 2.42 | 0.2665198 | 4.922 | 83560 | 2.39349E-10 | 2.79107E+13 | 2.3322E+18 | 0.03716 | 3105.103 |
|  |  |  |  |  |  | ∑= | 2.69981E+14 | 6.2761E+19 |  | 275378.1^b^ |

**Table S2.** Calculating the number (M_n_) and weight average molecular weight (M_w_) of EtGIPL1a. Twenty mg of EtGIPL1a were loaded onto BioSEC-3 at a flow rate of 1.2 mL/min. Vo is the void volume or Rt volume of Dextran Blue 2000 = 5.26 mL. Vc = 1 Column volume = 14.34 mL. Therefore, Vc-Vo = 9.08 mL.

^a^Molecular weight peak maxima (M_p_) = 216271 g/mol.

^b^Weight average molecular weight (M_w_) = 275378.1 g/mol.

Number average molecular weight (M_n_) = 232465.3 g/mol. (M_n_ = ΣniMi/Σni)

Polydispersity index = 1.184 (PDI = M_w_/M_n._).

|  | **EtGIPL1a** | | **EtISPFa** | |
| --- | --- | --- | --- | --- |
| **Monosaccharide residue** | **Mass (µg)** | **Mol %** | **Mass (µg)** | **Mol %** |
| Glucose (Glc) | 90.8 | 54.3 | 118 | 66.2 |
| Galactose (Gal) | 32.7 | 19.6 | 11.4 | 6.4 |
| Mannose (Man) | 18.6 | 11.1 | 11.9 | 6.7 |
| Fucose (Fuc) | 15.7 | 10.3 | 2.9 | 1.8 |
| Glucuronic acid (GlcA) | 7.3 | 4.0 | 19.4 | 10.1 |
| Rhamnose (Rha) | 0.9 | 0.6 | 5.0 | 3.1 |
| Xylose (Xyl) | - | - | 8.3 | 5.6 |
| Arabinose (Ara) | - | - | 0.4 | 0.2 |
| Total = | 165.9 | 100 | 177.2 | 100 |

**Table S3.** Monosaccharide composition of EtGIPL1a and EtISPFa. The results of EtISPFa had been previously reported^1^.

**References**

1. Zeb, M. *et al*. Structural elucidation and immuno-stimulatory activity of a novel polysaccharide containing glucuronic acid from the fungus *Echinodontium tinctorium*. *Carbohydr. Polym.* **258,** 117700 (2021).
